# Routine cine-CMR segmentation via a novel automated algorithm (LV-METRIC) for assessment of aortic physiology: a clinical validation study

**DOI:** 10.1186/1532-429X-17-S1-P387

**Published:** 2015-02-03

**Authors:** Parmanand Singh, Noel C Codella, Yi Wang, Zaid I Almarzooq, Nisha Bavalia, Grace Malonga, Steven M Markowitz, Mary J Roman, Richard B Devereux, Jonathan W Weinsaft

**Affiliations:** 1 Medicine- Cardiology, Weill Cornell Medical College, New York, NY, USA; 2 IBM T.J. Watson Research Center, New York, NY, USA

## Background

Routine cine-CMR is widely used to assess cardiac structure and function. Partial voxel interpolation has been shown to yield improved agreement with phantom derived chamber volumes and necropsy evidenced LV mass; the utility of partial voxel interpolation for assessment of aortic physiology has never before been tested.

## Methods

Cine-CMR (SSFP) was performed on 1.5 Tesla (GE) scanners; pulse sequence parameters were equivalent to those for routine CMR (typical TR 3.4 msec, TE 1.14 msec, flip angle 60^ο^, temporal resolution 30 msec). Images were acquired in conventional cardiac (2, 3, 4 chamber) long axis or axial imaging planes. Aortic area was uniformly measured in a non-aneurysmal location within the mid-descending thoracic aorta: Cine-CMR was quantified via a novel "partial voxel" segmentation algorithm (LV-METRIC) that accounts for relative proportion of blood within each individual imaging voxel. Maximum (systolic) and minimum (diastolic) aortic areas and brachial pulse pressure were used to calculate distensibility, a measure of arterial compliance, of the mid-descending thoracic aorta.

## Results

**Figure 1:**
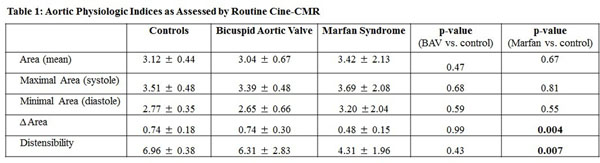
Aortic physiologic indices as assessd by routine cine-CMR

32 subjects were studied, among whom 22 had genetically-mediated aortopathies (13 bicuspid aortic valve [BAV], 9 Marfan syndrome [MFS]) and 10 were normative controls. Aortopathy subjects were similar to controls in age, gender, pulse pressure and body size (all p=NS). Aortic indices, compared between MFS, BAV and control groups are shown in Figure 1. As shown, absolute aortic size (measured in a non-aneurysmal region) was similar between groups (all p=NS). Dynamic change in aortic area (Δ Area) was lesser among MFS vs. controls (p=0.004) as well as BAV (p=0.03), but not between BAV vs controls (p=0.99). Aortic distensibility, as measured in all aortopathy subjects and 6 normative controls, demonstrated lower values among MFS subjects as compared to normative controls (p=0.007), with a similar trend when MFS and BAV groups were compared (p=0.08). There was no significant difference in distensibility between BAV vs controls (p=0.43).

## Conclusions

Routine cine-CMR can discern altered aortic physiology in non-aneurysmal regions in subjects with MFS. Larger longitudinal studies are needed to further evaluate the prognostic utility of cine-CMR segmentation, including use of central aortic blood pressure, as a potential biomarker of early aortic disease.

## Funding

Not applicable.

